# Monocyte Chemoattractant Protein-1 (MCP-1) Regulates Macrophage Cytotoxicity in Abdominal Aortic Aneurysm

**DOI:** 10.1371/journal.pone.0092053

**Published:** 2014-03-14

**Authors:** Qiwei Wang, Jun Ren, Stephanie Morgan, Zhenjie Liu, Changlin Dou, Bo Liu

**Affiliations:** 1 Division of Vascular Surgery, Department of Surgery, University of Wisconsin-Madison, Wisconsin, United States of America; 2 Luye Pharma Group, Yantai, China; Albert Einstein College of Medicine, United States of America

## Abstract

**Aims:**

In abdominal aortic aneurysm (AAA), macrophages are detected in the proximity of aortic smooth muscle cells (SMCs). We have previously demonstrated in a murine model of AAA that apoptotic SMCs attract monocytes and other leukocytes by producing MCP-1. Here we tested whether infiltrating macrophages also directly contribute to SMC apoptosis.

**Methods and Results:**

Using a SMC/RAW264.7 macrophage co-culture system, we demonstrated that MCP-1-primed RAWs caused a significantly higher level of apoptosis in SMCs as compared to control macrophages. Next, we detected an enhanced Fas ligand (FasL) mRNA level and membrane FasL protein expression in MCP-1-primed RAWs. Neutralizing FasL blocked SMC apoptosis in the co-culture. *In situ* proximity ligation assay showed that SMCs exposed to primed macrophages contained higher levels of receptor interacting protein-1 (RIP1)/Caspase 8 containing cell death complexes. Silencing RIP1 conferred apoptosis resistance to SMCs. In the mouse elastase injury model of aneurysm, aneurysm induction increased the level of RIP1/Caspase 8 containing complexes in medial SMCs. Moreover, TUNEL-positive SMCs in aneurysmal tissues were frequently surrounded by CD68^+^/FasL^+^ macrophages. Conversely, elastase-treated arteries from MCP-1 knockout mice display a reduction of both macrophage infiltration and FasL expression, which was accompanied by diminished apoptosis of SMCs.

**Conclusion:**

Our data suggest that MCP-1-primed macrophages are more cytotoxic. MCP-1 appears to modulate macrophage cytotoxicity by increasing the level of membrane bound FasL. Thus, we showed that MCP-1-primed macrophages kill SMCs through a FasL/Fas-Caspase8-RIP1 mediated mechanism.

## Introduction

Abdominal aortic aneurysm (AAA) is a common, progressive, and life-threatening degenerative vascular disease. AAA is histologically characterized by transmural infiltration of inflammatory cells, depletion of vascular smooth muscle cells (SMCs), and degradation of arterial extracellular matrix (ECM) [1], [2]. Data generated in mouse aneurysm models indicate that macrophage-mediated inflammation is critical for the development and progression of aneurysm [3], [4]. Depletion of macrophages [5] or preventing them from expressing ECM degrading enzymes such as MMPs [6]–[8] protected mice from developing aneurysm. Monocyte chemoattractant protein-1 (MCP-1) has been implicated in the pathogenesis of several cardiovascular diseases including AAA. Elevated MCP-1 mRNA and protein expression has been consistently detected in aneurysmal aortic tissues of human patients as well as animal models [9]–[12]. Blocking MCP-1 signaling either through genetic deletion or siRNA-mediated knockdown, or through inhibition of its receptor, CCR2, successfully inhibits AAA development [3], [13], [14]. We have previously demonstrated that ectopic administration of recombinant MCP-1 to the arterial wall of PKCδ^−/−^ mice, which has an aneurysm-resistant phenotype, was sufficient to restore local inflammatory response and AAA development [15]. MCP-1 has long been known to be a major chemotactic cytokine, and recent studies demonstrated that MCP-1 also contributes to disease progression through regulating genes that can cause cell death or differentiation [16]. Moreover, several groups have shown MCP-1′s cytotoxic effect(s) to be mediated through activation of macrophages [17], [18]. In mixed cultures containing photoreceptors and macrophages, an increasing MCP-1 concentration correlated to an increase in photoreceptor death, though MCP-1 showed no direct effect on photoreceptor survival after depleting macrophages from the cultures [17].

Macrophage's cytotoxic function has also been documented in models of cardiovascular diseases. In atherosclerosis, macrophages were found to induce SMC apoptosis, thus contributing to plaque rupture [19], [20]. Furthermore, in a model of heart failure, macrophages were reported to induce cell apoptosis through secretion of Fas Ligand [21]. Although several cytokines produced by macrophages including TNFα and IL-1β are capable of inducing cell death, whether infiltrating macrophages directly contribute to SMC depletion remains to be explored in aneurysm.

In this study, we tested the hypothesis that infiltrating macrophages directly cause death of residential SMCs. Our data suggests that MCP-1-primed macrophages elicit aortic SMC apoptosis through a FasL/Fas-Caspase-8-RIP1 mediated pathway.

## Materials and Methods

### Ethics statement

All animal experiments in this study were approved by the Institutional Animal Care and Use Committee at the University of Wisconsin-Madison (Protocol M02284) and performed in accordance with the Guide for the Care and Use of Laboratory Animals published by the United States National Institutes of Health.

### Animal model

C57BL/6J mice and MCP-1 knockout mice were purchased from The Jackson Laboratory (Bar Harbor, Maine). Male mice aged 12∼14 weeks, underwent an elastase-induced AAA model as described previously [2], [8], [11]. Briefly, after placing temporary ligatures around the proximal and distal aorta, an aortotomy was created at the bifurcation. A heat-tapered segment of PE-10 polyethylene tubing (Baxter Healthcare Corp., Deerfield, IL) was introduced through the aortotomy and secured. 0.45 U/mL type I porcine pancreatic elastase (Sigma, St. Louis, MO) was administrated through the tubing and allowed to incubate for 5 min at a constant pressure of 100 mm Hg. As a control, a separate group of mice were treated with equal concentration of heat-inactivated (100°C) elastase for 5 min.

Mice that underwent surgery were anesthetized: inhaled isoflurane via a chamber at 4% was initially used, followed by a mask with 2% of isoflurane mixed with 100% oxygen. Buprenorphine was administered subcutaneously at a dose of 0.05 mg/kg immediately after surgery. Subsequently, a 2.5% Xylocaine topical ointment was applied to the suture site. Additional doses of Buprenorphine were given via intraperitoneal injection every 8–12 hours after surgery for the first 48 hours. Mice were euthanized with 100% oxygen/5% isoflurane.

### General materials

Dulbecco's Modified Eagle Medium (DMEM) was from Gibco (Life Technologies, Carlsbad, CA). Primary antibodies used include anti-cleaved caspase-8, anti-cleaved caspase-9, anti-cleaved caspase-3, anti-cleaved PARP, anti-β-actin (Cell Signaling Technologies, Danvers, MA), anti-Fas Ligand, anti-alpha smooth muscle Actin antibody (Abcam, Cambridge, MA), anti-CD68 (AbD Serotec, Raleigh, NC), Rat Anti-Mouse Fas Ligand Monoclonal Antibody for neutralization, Rat IgG1 Isotype Control (R&D Systems, Minneapolis, MN), Anti-Mouse Fas Ligand FITC, Armenian Hamster IgG Isotype Control FITC (eBioscience, San Diego, CA). Fluorophore-conjugated secondary antibodies and 4′6-diamidino-2-phenyl-indole, dihydrochloride (DAPI) were purchased from Molecular Probes (Life Technologies, Carlsbad, CA). Horseradish Peroxidase (HRP)-conjugated Antibodies were purchased from Bio-Rad (Hercules, CA). *In Situ* Cell Death Detection Kit was from Roche Applied Science (Indianapolis, IN). siRNAs used in this study were Silencer Select siRNAs from Ambion (Life Technologies, Carlsbad, CA). Other chemicals and reagents if not specified were purchased from Sigma-Aldrich (St. Louis, MO).

### Cell culture and priming macrophages with MCP-1

Primary mouse aortic SMCs were isolated from the thoracic and abdominal aorta as described by Clowes et al [22], [23]. Briefly, the aorta was isolated from the aortic arch to the iliac bifurcation and incubated 30 minutes in digestion buffer (DMEM, Bovine serum albumin, Collagenase, Soybean trypsin inhibitor, and Elastase Type III) at 37°C. Adventitia was pulled away from the medial layer; tissues were minced, and further digested for 4 hours at 37°C. Tissue was spun to a pellet by centrifugation and washed with 10% fetal bovine serum (FBS; Gemini, Woodland, CA) DMEM once, before suspension in a small volume of 10% FBS DMEM and left undisturbed for 48 hours to allow cells to migrate from tissue. Primary SMCs were grown at 37°C in 5% CO_2_ in DMEM modified to contain 4 mM L-Glutamine, 1 g/L D-Glucose, and 110 mg/L Sodium Pyruvate (Life Technologies, Carlsbad, CA) supplemented with 10% fetal bovine serum (FBS) and antibiotics. Cells between three and seven passages were used. The murine macrophage cell line RAW264.7 cells were obtained from American Type Culture Collection (ATCC, Manassas, VA) and grown as recommended in DMEM modified containing 4.5 g/L D-Glucose supplemented with 10% FBS and antibiotics.

To prime macrophages, RAWs were made quiescent by incubation in medium containing 0.5% FBS for 24 hours and then treated with 100 ng/ml MCP-1 (R&D Systems, Minneaspolis, MN) for 24 hours. To set up a co-culture system, MCP-1-primed or PBS-treated RAWs were collected and subcultured onto SMCs in the six-well plate in a 1:1 ratio.

### Flow cytometry assay for apoptosis

Assays were carried out by using an Annexin V-PE/7-AAD Apoptosis detection Kit (BD Biosciences, San Jose, CA). Cultures were rinsed with ice-cold PBS and incubated with accutase (Life Technologies, Carlsbad, CA) at 37°C for 2 min. The detached cells (from culture medium, PBS wash, and accutase treatment) were collected by centrifugation (2000 rpm, 5 min). The combined cell pellets were further washed twice with ice-cold PBS and resuspended in 100 μl binding buffer from the apoptosis detection Kit. Resuspended cells were then incubated 5 μl PE Annexin-V and 5 μl 7-AAD in dark at room temperature for 15 min. After incubation, 400 μl binding buffer was added to each sample. Cells were analyzed using a Becton Dickinson Biosciences FACSCalibur (BD Biosciences, San Jose, CA).

### RNA Isolation and Quantitative Real-Time PCR (qRT-PCR)

Total RNA was isolated from cultured cells using RNeasy Plus Mini Kit (Qiagen, Valencia, CA), or using Trizol reagent (Life Technologies, Carlsbad, CA) according to manufacturer's protocols. Then, two microgram total RNA was used for the first-strand cDNA synthesis (Applied Biosystems, Carlsbad, CA). qRT-PCR was carried out using the 7500 Fast Real-Time PCR System (Applied Biosystems, Carlsbad, CA). Each cDNA template was amplified in triplicate using SYBR Green PCR Master Mix (Applied Biosystems, Carlsbad, CA) with gene specific primers for RIP1, FasL and GAPDH, which were purchased from Qiagen (Valencia, CA). The relative mRNA levels were calculated using the 2^−ΔΔCt^ method and normalized by the level of GAPDH.

### Immunoblotting

Primary mouse aortic SMCs were seeded to a six-well plate and cultured. RAWs were made quiescent by incubation in medium containing 0.5% FBS for 24 hours and then treated with 100 ng/ml MCP-1 (R&D Systems, Minneapolis, MN) for 24 hours. MCP-1-primed RAWs were collected and subcultured onto SMCs in the six-well plate in a 1:1 ratio. Cells were lysed in RAPI buffer (Sigma-Aldrich, St. Louis, MO). Equal amounts of protein extract were separated by SDS-PAGE and transferred to polyvinylidene fluoride (PVDF) membranes. The membranes were then incubated with primary antibodies overnight at 4°C, followed by HRP-labeled secondary antibodies. Labeled proteins were visualized with an enhanced chemiluminescence system (PerkinElmer-cetus, Boston, MA). For quantification, optical density of proteins determined by ImageJ (National Institute of Health, Bethesda, MD) was normalized to the β-actin protein density.

### Immunohistochemistry

Tissues were perfusion-fixed with a mixture of 4% formaldehyde in phosphate buffered saline (PBS) and were imbedded in O.C.T. Compound (Sakura Tissue Tek, Netherlands). All frozen sections were cut to 6 μm thick using a Leica CM3050S cryostat, and were permeabilized with 0.1% Triton X-100 in Tris-buffered saline (TBS) for 10 minutes at room temperature. Non-specific sites were blocked using 1% bovine serum albumin (BSA), 10% normal donkey serum in TBS for 2 hours at room temperature. All primary antibodies diluted in TBS with 1% BSA were then applied onto arterial sections, and incubated overnight at 4°C. On the second day, arterial sections were rinsed with TBS plus 0.025% Triton X-100 with gentle agitation for twice and five minutes each time. And then the arterial tissues were incubated with fluorophore-conjugated secondary antibodies diluted in TBS with 1% BSA for 1 hour at room temperature. Arterial sections were then rinsed with TBS for three times and five minutes each time. DAPI was used to stain the nuclei. Staining was visualized with a Nikon Eclipse Ti inverted microscope system and digital images were acquired using a Nikon DS-Ri1 digital camera. Nikon A1RSi Confocal system was used to take confocal images.

TUNEL staining and semi-quantification analysis of TUNEL-positive cells in the media of aneurysmal tissues were performed as previously described [15]. TUNEL-positive cells were counted in a blind fashion. The area of tunica media was measured with ImageJ (National Institute of Health, Bethesda, MD).

### *In Situ* Proximity Ligation Assay (PLA)

*In situ* proximity ligation assay (PLA) was performed to detect protein–protein interactions using Duolink In Situ Red Starter Kit according to the manufacturer's protocol (Olink Bioscience, Uppsala, Sweden). Briefly, cells were fixed with 4% paraformaldehyde at room temperature for 10 min followed by cell-membrane permeabilization with 0.02% Triton X-100 in TBS for 10 min. Tissue sections were prepared as described previously. The cells or arterial sections were then blocked with 1% BSA and 10% normal donkey serum in TBS for 2 hours at room temperature, and incubated with the indicated primary antibody pairs overnight at 4 °C. Oligonucleotide-conjugated secondary antibodies (PLA probe MINUS and PLA probe PLUS) against each of the primary antibodies were applied, and ligation as well as amplification was carried out to produce rolling circle products. These products were detected with fluorescent labeled oligonucleotides and the samples were counterstained using Duolink Mounting Medium with DAPI. Fluorescence was visualized with a Nikon Eclipse Ti inverted microscope system and digital images were acquired using a Nikon DS-Ri1 digital camera

### Statistical analysis

Data are presented as mean±SEM. Statistical differences were evaluated by two-tailed Student's *t*-test or one-way ANOVA with Bonferroni's post-hoc test in the case of multiple comparisons. Differences with *p*-value <0.05 were considered statistically significant. All experiments were repeated at least three times.

## Results

### MCP-1 Primed-Macrophages Show Higher Cytotoxicity

**Figure 1:**
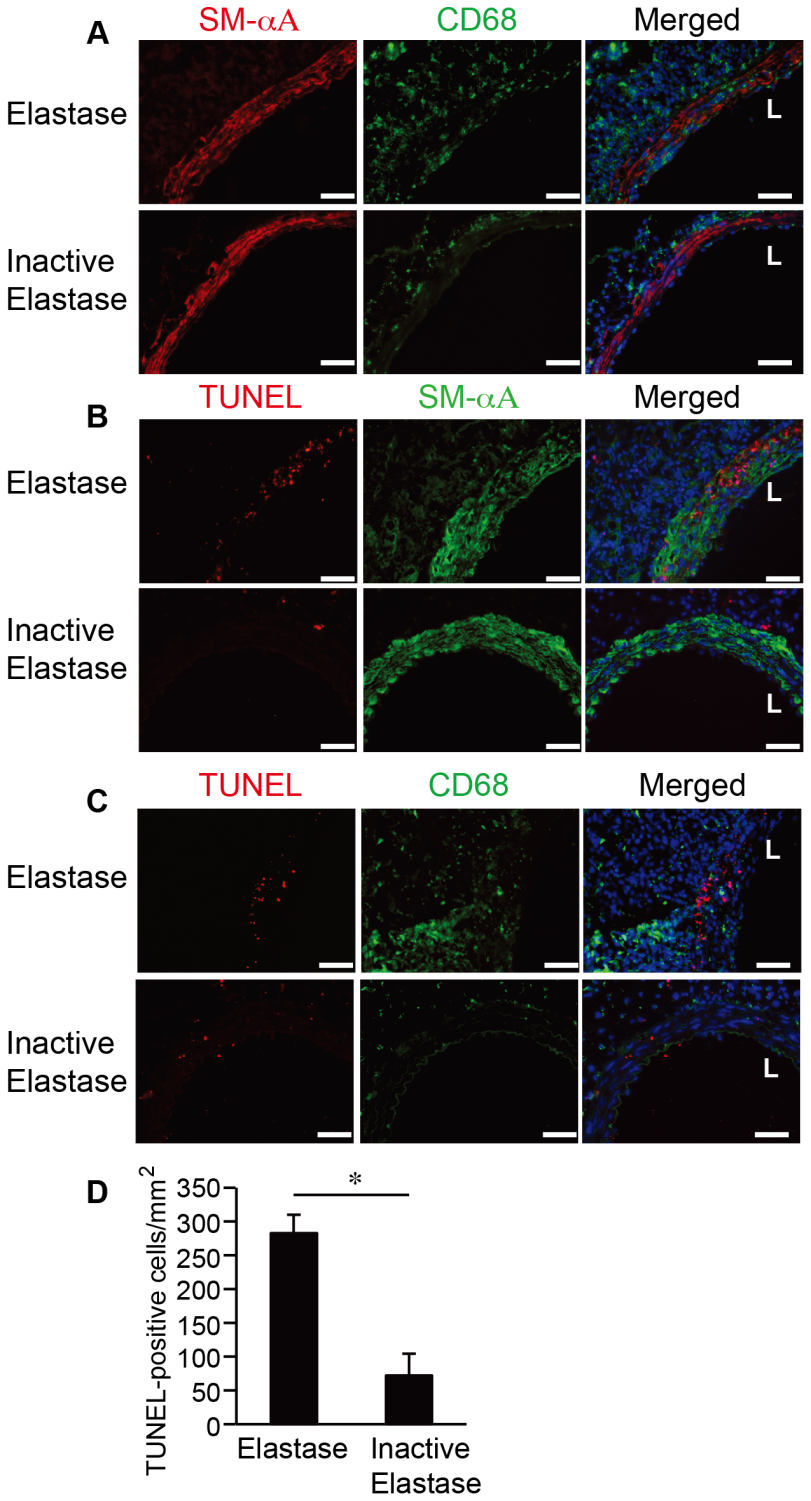
Medial accumulation of macrophages coincides with SMC apoptosis in elastase-induced mouse AAA. Mice were subjected to aneurysm induction by elastase. Inactive elastase-treated arteries were used as control. Aortas were harvested 3 days after surgery and double stained for (**A**) SMCs (SM-αA, red) and macrophages (CD68, green), or for (**B**) apoptotic cells (TUNEL, red) and SMCs (SM-αA, green), or for (**C**) apoptotic cells (TUNEL, red) and macrophages (CD68, green). Sections were counterstained with DAPI (blue). L indicates Lumen. Scale bar, 50 μm. Magnification, 40X. (**D**) Quantification of TUNEL-positive cells in the media of elastase or heat-inactivated elastase injured arteries on day 3 after surgery. Data are mean ± SEM. n = 3, *p<0.05, Two-tailed Student's *t*-test.

Double immunostaining of murine aneurysmal aortic tissues demonstrated the elevation of SMC apoptosis and accumulation of macrophages in the media of elastase-treated arteries (**Fig. 1** **A-D**). Histological examination of aneurysmal tissue sections confirmed elevated levels of MCP-1 (**Fig. S1**). Using RAW264.7 cells, a mouse monocyte/macrophage cell line, we tested whether MCP-1 changes macrophage functions. By quantitative PCR (qPCR), we showed that MCP-1 (100 ng/ml) enhanced expression of a pro-inflammatory cytokine TNFα that is known to associate with macrophage M1 phenotype, but reduced the expression of arginase I, a marker for the anti-inflammatory M2 phenotype (**Fig. S2**).

**Figure 2:**
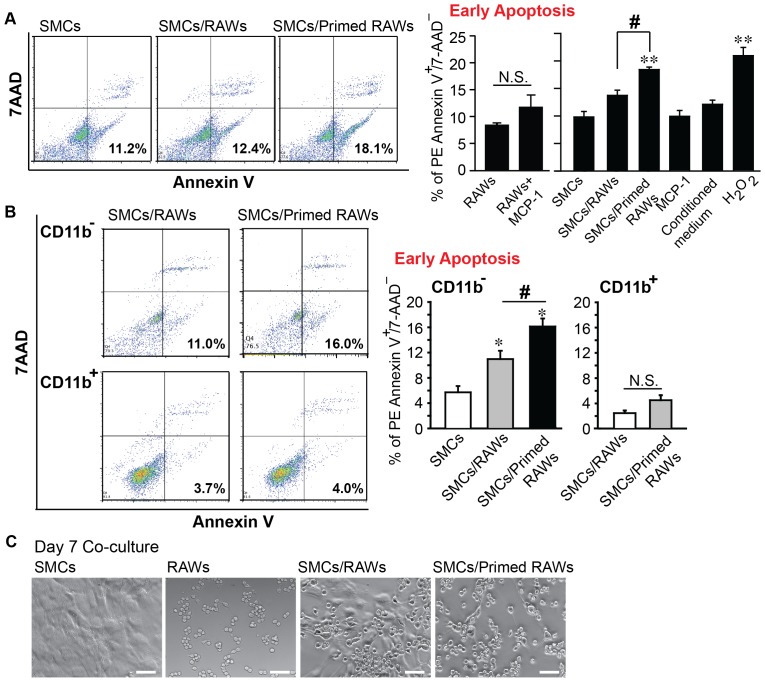
MCP-1-primed macrophages show higher cytotoxicity. (**A**) RAW macrophages (after 24 hours starvation) and SMCs were exposed to various culture conditions with or without addition of MCP-1 (100 ng/ml) for 24 hours. SMCs were also co-cultured with MCP-1-primed RAWs (100 ng/ml, 24 h), or naïve RAWs (PBS treated), or cell-free conditioned medium harvested from MCP-1-primed RAWs for 24 hours. Early apoptotic cells were identified as PE Annexin-V^+^/7-AAD^−^ by flow cytometric analysis. H_2_O_2_ (700 μM, 4 h) treatment was used as a positive control. (**B**) Co-culture was stained with the monocyte/macrophage marker CD11b prior to PE Annexin-V/7-AAD staining. (**C**) Representative bright field images of SMCs, RAWs, SMCs/RAWs co-culture (7 days), and SMCs/MCP-1 primed RAWs co-culture (7 days). Scale bar, 50 μm. Magnification, 20X. Data are mean±SEM. n = 3∼6, *p<0.05, **p<0.01 vs. untreated SMCs, #*p*<0.05, N.S.  =  not significant, One-way ANOVA.

To study whether MCP-1 influences the pro-apoptotic capability of macrophages, we set up a co-culture system containing mouse aortic SMCs and RAW264.7. Using flow cytometric analysis, we compared cell apoptosis (PE Annexin V^+^/7-AAD^-^) after exposing SMCs to various culture conditions with or without addition of MCP-1 (100 ng/ml), or MCP-1-primed RAWs (100 ng/ml, 24 h), or naïve RAWs (PBS treated), or cell-free conditioned medium harvested from MCP-1-primed RAWs. As shown in **Fig. 2** **A**, MCP-1 alone did not significantly change apoptosis in RAWs (11.833%±2.216% vs. 8.488%±0.413%, *p* = 0.23). Co-culturing SMCs with the naïve RAWs slightly increased apoptosis, however, this trend failed to reach statistical significance based on One-Way ANOVA. In contrast, the apoptosis level was significantly higher in the co-culture with MCP-1-primed RAWs (18.425%±0.450%) comparing to SMCs alone or coculture with naïve RAWs (9.817%±0.992% and 13.725%± 0.718%, respectively). Neither MCP-1 alone nor cell-free conditioned media significantly altered apoptosis compared to untreated SMCs, suggesting cell-cell contact is required for apoptosis induction in the co-culture.

Using CD11b as a monocyte/macrophage marker, we sought to identify apoptotic cells in the co-culture. We found less than 20% of apoptotic cells to be CD11b^+^ RAWs (data not shown), and the level of RAW apoptosis was not altered by the presence or absence of MCP-1 (**Fig. 2B**). In contrast, 10.977%± 1.321% of CD11b^-^ cells, likely SMCs, were apoptotic, which was further elevated to 16.133%±1.255% (*p*<0.05) by MCP-1-primed RAWs. Bright field images showed abnormal appearance of the few remaining SMCs in SMCs/MCP-1-primed RAWs co-culture (**Fig. 2C**).

### Activation of Caspase 8/Caspase 3 pathway in co-culture

**Figure 3:**
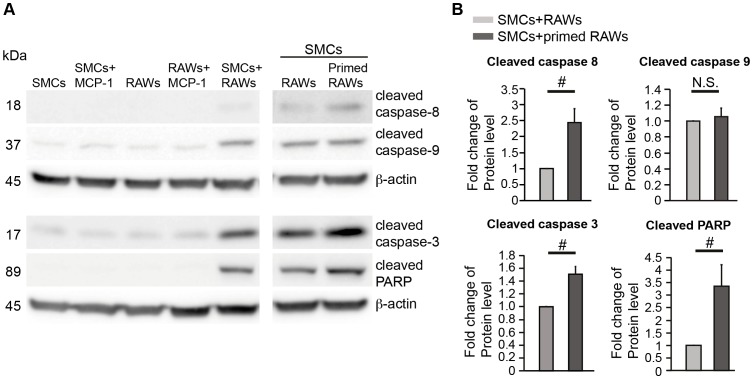
Activation of Caspase 8/Caspase 3-mediated cell death pathway in the co-culture. (**A**) Representative immunoblots of cleaved caspase-8, -9, -3, and –PARP in SMCs, RAWs, and co-cultures as indicated. RAWs were primed by treatment of MCP-1 (100 ng/ml) for 24 h prior to co-culture. Cells were harvested after 3 days of co-culture or individual culture. (**B**) Quantifications of Western blots for cleaved caspase-8, -9, -3, and -PARP from SMCs co-cultured with naïve or MCP-1 (100 ng/ml, 24 h) primed RAWs. Data are mean±SEM. n = 3∼6, #*p*<0.05, N.S.  =  not significant, Two-tailed Student's *t*-test.

Next, we explored the apoptotic pathway(s) by examining activation of major caspases in the co-culture. Western blot analysis revealed that exposing SMCs to MCP-1-primed RAWs significantly increased levels of cleaved caspase 8, cleaved caspase 3, and cleaved PARP compared to naïve RAWs (**Fig. 3**), while the level of cleaved caspase 9 was not dependent upon MCP-1 priming of RAWs present in co-culture. Taken together, our data indicate that SMC apoptosis triggered by MCP-1 primed RAWs is mediated via an extrinsic apoptotic pathway, which involves caspase 8 and 3 but not caspase 9.

### MCP-1 enhanced the pro-apoptotic capacity of RAWs by increasing Fas ligand (FasL) expression

**Figure 4:**
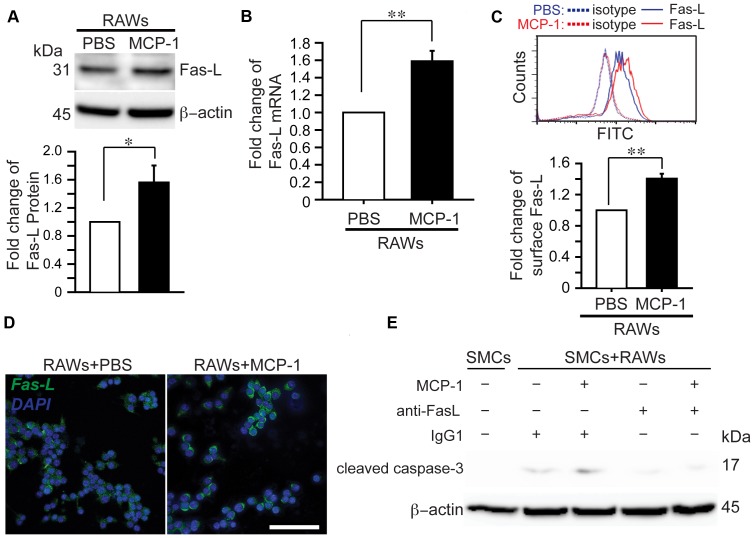
MCP-1 enhances the pro-apoptotic capacity of RAWs by increasing membrane Fas ligand expression. (**A**) Western blot analysis of total FasL protein from naïve RAWs or MCP-1 (100 ng/ml, 24 h) primed RAWs. (**B**) Real-time PCR analysis of total FasL mRNA level. (**C**) Flow cytometric analysis of FasL protein expression on non-permeabilized RAWs to evaluate the membrane FasL. (**D**) Confocal images of Fas-L (Green), nuclei (DAPI, blue) of naïve RAWs or MCP-1 primed RAWs, Scale bar, 50 μm. Magnification, 40X. (**E**) Immunodepletion of FasL with a neutralizing anti-FasL antibody attenuated apoptosis in the co-culture. Data are mean±SEM. n = 3∼6. *p<0.05, **p<0.01. Two-tailed Student's *t*-test.

To understand how MCP-1 regulates the cytotoxic capacity of macrophages, we turned to the Fas/FasL pathway that is known to work through the extrinsic apoptotic pathway and has also been implicated in the pathogenesis of aneurysm [24], [25]. Treating RAWs with MCP-1 (100 ng/ml, 24 h) caused a significant increase in levels of FasL protein level (1.56±0.228 fold, *p*<0.05) and mRNA expression (1.59±0.118 fold, *p*<0.01) (**Fig. 4** **A, B**). Since membrane FasL (memFasL) rather than secreted FasL is essential for cytotoxic activity [24], [26], we further compared memFasL levels between MCP-1-primed and naïve RAWs. Using flow cytometry to detect FasL on non-permeabilized RAWs, we showed that MCP-1 significantly increased levels of memFasL (**Fig. 4C**). Immunofluorescence confocal microscopy further supported that MCP-1-primed RAWs displayed more abundant memFasL (**Fig. 4D**). Moreover, we repeated the co-culture study in the presence or absence of neutralizing anti-FasL antibody. Using level of cleaved caspase 3 as a readout for apoptosis, we demonstrated that immunodepletion of FasL abolished the effects of MCP-1 on macrophages cytotoxicity in the co-culture (**Fig. 4E**).

### RIP1 underlies SMC apoptosis

**Figure 5:**
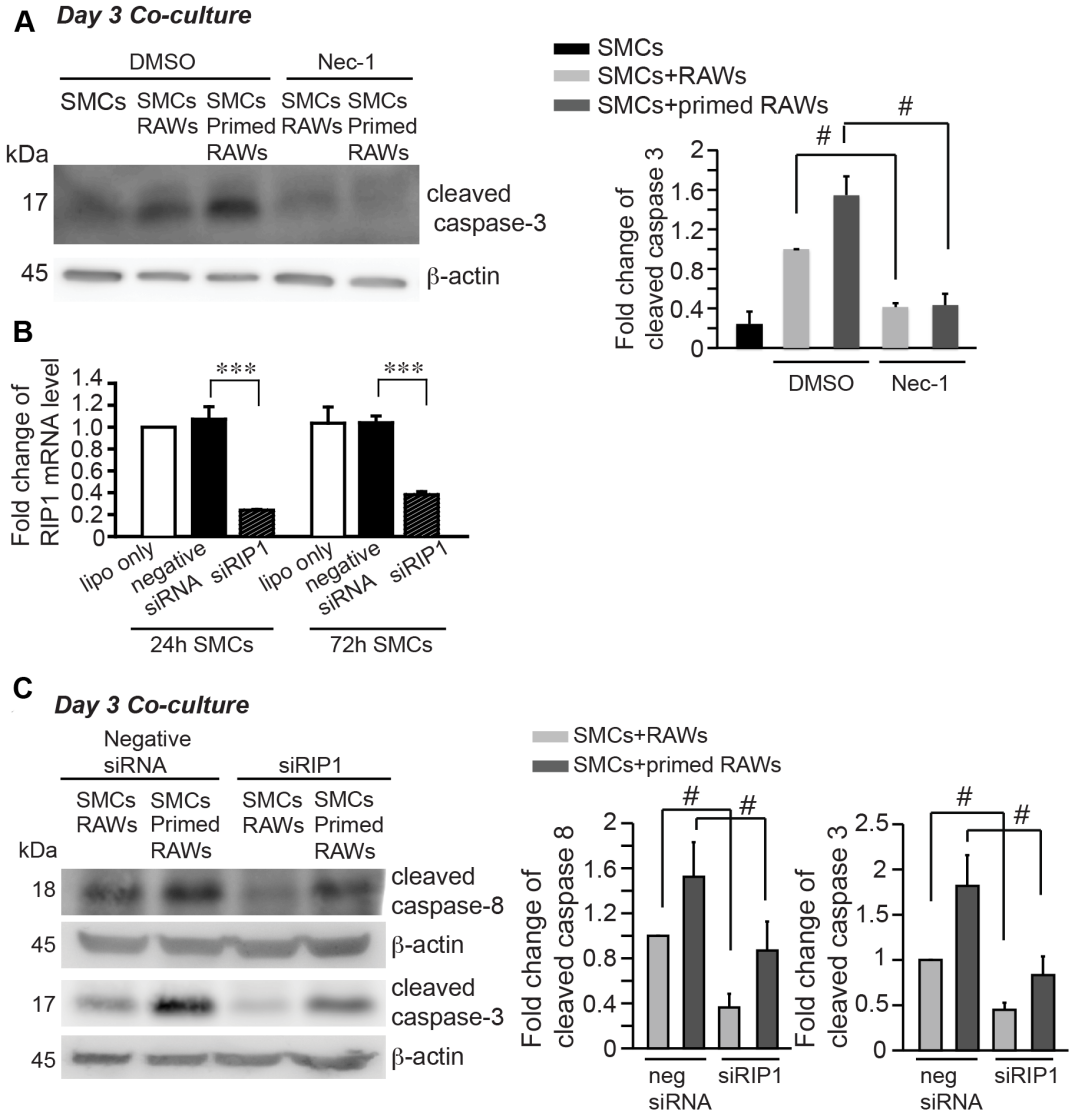
Receptor interacting protein-1 (RIP-1) underlies SMC apoptosis in the co-culture. (**A**) Inhibition of RIP1 with necrostatin-1 (nec-1, 40 μM) profoundly diminished apoptosis in the co-culture. (**B**) Efficiency of siRNA-mediated knockdown of RIP1 was examined by real-time PCR analysis at 24 hours and 72 hours after siRNA transfection to SMC. (**C**) SiRNA-mediated knockdown of RIP1 in SMCs prior to exposing SMCs to macrophages significantly attenuated apoptosis in the co-culture. Apoptosis was evaluated by Western blot analysis of cleaved caspase-8 and -3. Data are mean±SEM. n = 3∼4, #*p*<0.05, ***p<0.001, Two-tailed Student's *t*-test (A and C), One-way ANOVA (B).

We next sought to investigate the signaling molecules downstream of FasL/Fas axis in macrophage-induced SMC apoptosis. Receptor interacting protein kinase 1 (RIP1) has been implicated in extrinsic apoptotic pathway and programed cell necrosis [26]–[28]. We first inhibited RIP1 kinase activity with a chemical inhibitor necrostatin-1 (nec-1, 40 μM), and found that nec-1 profoundly diminished apoptosis in the co-culture (**Fig. 5A**). Then, we conducted siRNA-mediated knockdown of RIP1 in SMCs prior to exposing SMCs to RAWs (with or without MCP-1 priming). Accordingly, both cleaved caspase 8 and cleaved caspase 3 were significantly decreased in the co-culture (**Fig. 5B, C**).

**Figure 6:**
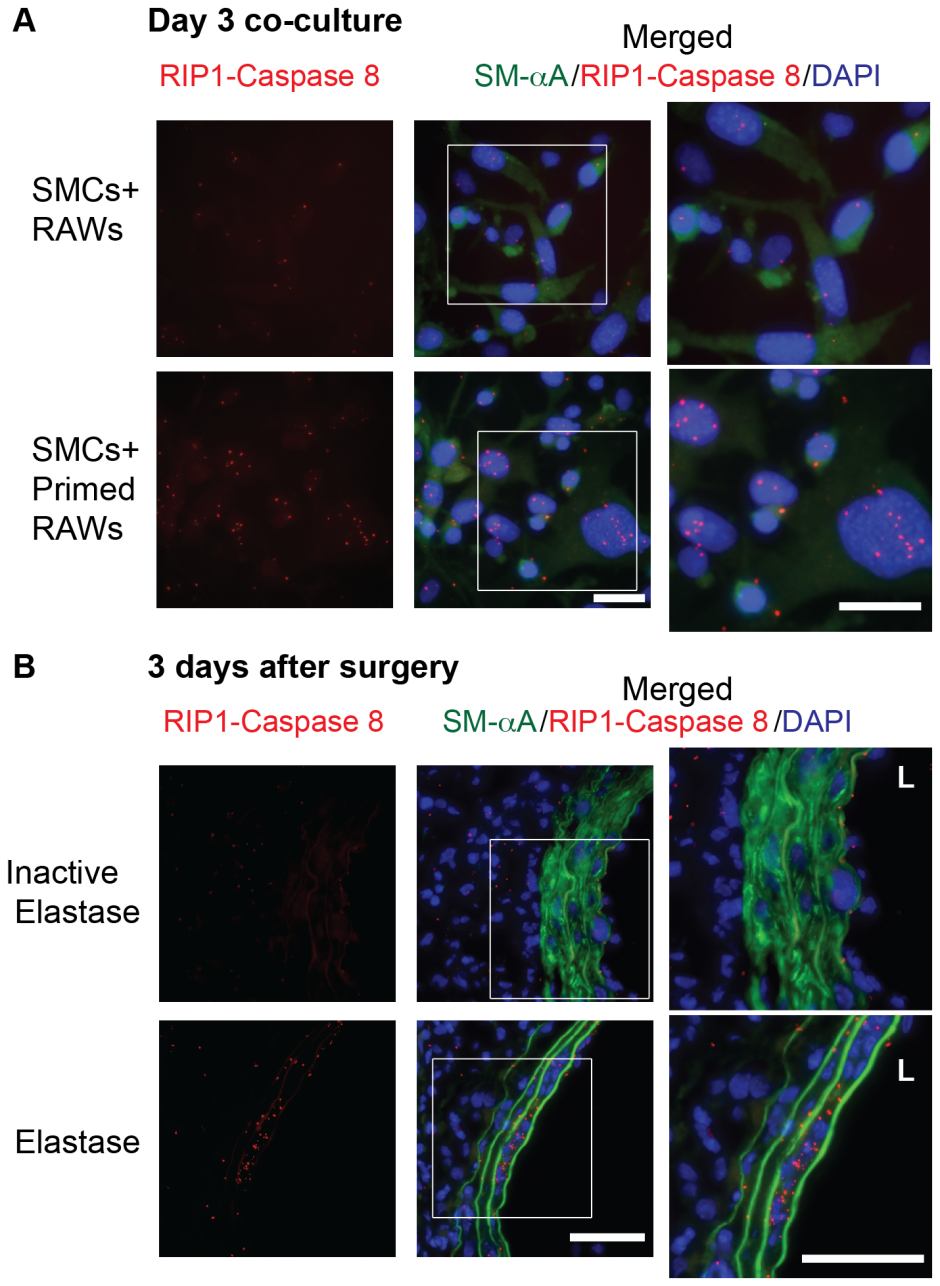
Increased RIP1/Caspase 8 containing complexes in SMCs exposing to primed RAWs and aneurysmal tissues. (**A**) SMCs were exposed to MCP-1 primed RAWs or naïve RAWs for 3 days. Left: RIP1/Caspase 8 containing complex formation was examined by in situ proximity ligation assay (PLA). Middle: co-immunostaining for SMCs (SM-αA, green) and RIP1/Caspase 8 containing complex (red spots). Nuclei (DAPI, blue). Right: magnified view of the boxed areas in the middle panel. Scale bar, 100 μm. (**B**) Increased RIP1/Caspase 8 containing complex in elastase-treated aortas. Arteries were harvested 3 days after aneurysm induction by elastase infusion. Heat-inactivated elastase served as a control. Nuclei (DAPI, blue), SMCs (SM-αA, green), RIP1/Caspase 8 containing complex (red spots). Higher magnified views of highlighted regions were shown on the right. L indicates lumen. Scale bar, 50 μm.

Furthermore, we examined a cell death complex containing RIP1 and Caspase 8 [26], [27]using the *in situ* proximity ligation assay (PLA), in which protein-protein interactions are visualized as fluorescent spots by rolling-circle amplification reactions dependent on the close proximity (<40 nm) of the target proteins [29]. Co-culture of SMCs, as identified by positive staining for smooth muscle α-actin (SM-αA), with MCP-1-primed RAWs exhibited increased levels of RIP1/Caspase 8 containing cell death complexes as compared to naïve RAWs (**Fig. 6A**). Next, we investigated RIP1/Caspase 8 containing cell death complexes in experimental AAA tissues. As shown in **Fig. 6B**, a very low level of RIP1/Caspase 8 containing complexes was detected in aorta treated with heat-inactivated elastase (control), particularly in SM-αA positive SMCs. This was in sharp contrast to the elastase-treated arteries, in which RIP1/Caspase 8 containing complexes were abundantly present in medial SMCs as well as in the adventitia.

### MCP-1 is necessary for FasL expression in aneurysm

**Figure 7:**
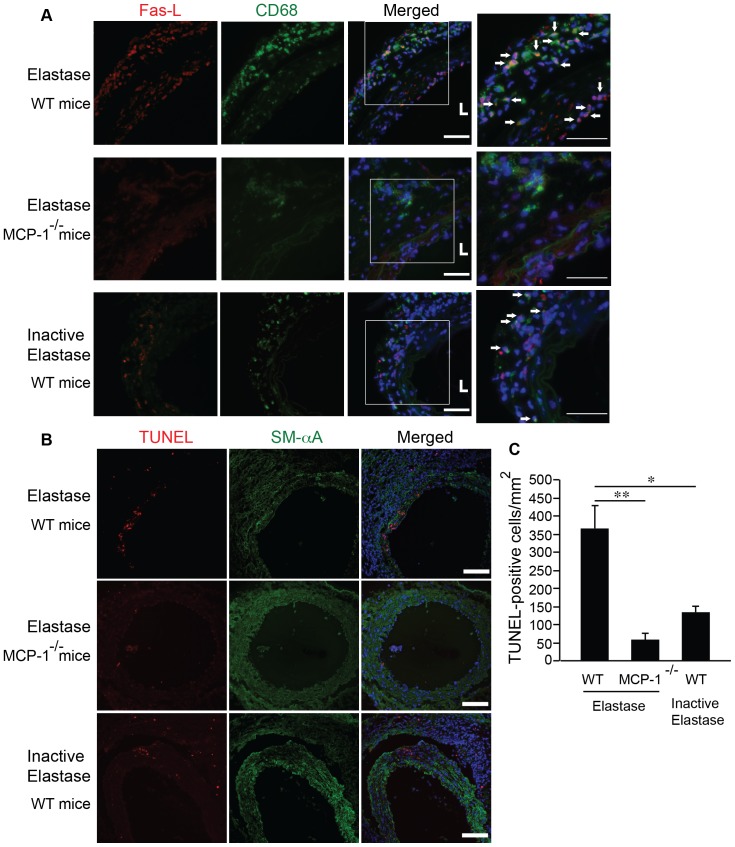
MCP-1 is necessary for FasL expression in infiltrated macrophage following aneurysm induction. (**A**) WT or MCP-1^−/−^ mice were subjected to aneurysm induction by elastase. Inactive elastase-treated arteries were used as control. Cross-sections harvested 3 days after surgery were stained for FasL (red) and macrophages (CD68, green). Fourth column represents increased magnification of highlighted region in the third column. Arrows indicate cells positive for both CD68 and FasL. L indicates lumen. Scale bar, 50 μm. (**B**) Cross-sections harvested 7 days after surgery were stained for TUNEL (red) and SMCs (SM-αA, green). Scale bar, 100 μm. (**C**) A semi-quantitative analysis of TUNEL-positive cells in the media of WT or MCP-1^−/−^ arteries on day 7 after elastase or heat-inactivated elastase infusion. Data are presented as mean ± SEM. n = 3, *p<0.05, **p<0.01, One-way ANOVA.

We subjected a strain of MCP-1 knockout mice to aneurysm induction using elastase-infusion model. Compared to the aorta infused with heat-inactivated elastase, elastase-infused wildtype aorta showed markedly higher number of macrophages in adventitial and medial layers (**Fig. 7A**). Furthermore, immunofluorescent co-stain demonstrated that the majority of FasL positive cells also expressed CD68, suggesting macrophages are a major source of FasL (**Fig. 7A**). Of note, not all FasL positive cells were also CD68 positive, indicating that macrophages are not the sole source of FasL. Nevertheless, MCP-1 gene deficiency inhibited macrophage infiltration as expected. However, macrophages were still detectable in the aortas of MCP-1 knockout mice following aneurysm induction, albeit at a reduced level. Among these infiltrated macrophages, none was stained positively for FasL expression (**Fig. 7A**). Additional arterial sections from different MCP-1 knockout mice were shown in Fig. S3. The absence of FasL staining in these arteries supports the critical role of MCP-1 in regulation of FasL expression during aneurysm formation. In contrast, in the elastase-treated WT aorta 46.9%±4.9% of infiltrated macrophages expressed FasL (**Fig. 7A**). Furthermore, MCP-1 knockout mice showed significantly diminished apoptosis as compared to WT mice following aneurysm induction (**Fig. 7B, C**).

## Discussion

Macrophages are the dominant type of inflammatory cells found in human and experimental AAA, and have been demonstrated to be critical mediators of aneurysm formation in different mouse models of AAA [4], [5], [30]. Macrophages are well-recognized sources of MMPs and proinflammatory cytokines during aneurysm development [6], [8], [31]. Depletion of circulating monocytes with clodronate-containing liposomes prevented aneurysm formation and preserved vascular SMCs in mice treated with AngII and anti-TGF-β antibody, suggesting a role of macrophages in reduction of SMC cellularity during AAA development [5]. Current data demonstrate that early macrophage infiltration to the SMC-rich medial layer coincided with SMC apoptosis in elastase-induced aneurysm, which is consistent with the observations in previous studies using the mouse model of Ang II–induced aneurysm [5], [32]. While macrophages produce several cytokines including TNFα and IL-1β that are pro-apoptotic, prior work showed that human blood-derived macrophages induced cell-cell contact-dependent vascular SMC apoptosis [33]. Similarly, in the current study, macrophage-conditioned media did not affect the viability of SMCs, which supports the necessity of cell-cell contacts at least in the context of MCP-1 mediated macrophage-induced apoptosis. Furthermore, TNFα was previously implicated in the promotion of human macrophage-induced vascular SMC apoptosis through a mechanism involving cooperative interactions with Nitric Oxide and FasL/Fas [20]. Here, our data demonstrate that FasL was required for SMC apoptosis induced by MCP-1-primed macrophages. This notion is further illustrated by diminished macrophage infiltration as well as FasL expression in MCP-1 knockout mice. Consistently, previous studies show that macrophages induced SMC apoptosis when macrophages/SMC ratios are high, and particularly when macrophages are differentiated or activated. Conversely, a low macrophage/SMC ratio is thought to trigger SMC proliferation by producing growth factors, particularly before macrophage differentiation or activation [19], [20], [34]. Given the crucial roles of cytokine(s) in modulation of macrophages differentiation [35], [36], it would be meaningful to investigate whether and which aneurysm-related cytokine(s) can modulate macrophage cytotoxicity, which may further cause SMC apoptosis in AAA.

MCP-1 as an important mediator of macrophage recruitment has been well documented in the pathogenesis of AAA as well as many other cardiovascular diseases [9], [13], [37]. Our data are in agreement with the emerging literature that suggests MCP-1 also plays critical roles in mediating apoptosis, either via a direct mechanism or through activation of macrophages [17], [18], [38]. By inducing oxidative stress through activation of macrophages, MCP-1 was shown to induce apoptosis of sensory neurons in the retina [17], [18]. By overexpressing MCP-1 in mouse cardiomyocytes, Kolattukudy and colleagues produced cardiac inflammation and develop heart failure [39]. The same investigative group later demonstrated that FasL released from infiltrating mononuclear cells plays a critical role in the manifestation of cell death and subsequent heart failure of MCP-1 transgenic mice [21]. In human monocytes MCP-1, through binding to CCR2, was found to induce the expression of a transcription factor called MCP-induced protein (MCPIP). Ectopic expression of MCPIP resulted in apoptosis in HEK293 cells as well as the cardiomyoblast cell line H9C2 [38]. Other functions of MCPIP as a deubiquitinase or RNase have also been described [16]. Whether MCPIP is involved in the FasL expression we observed in MCP-1-primed macrophages remains to be investigated.

Vascular SMCs can be an important source of inflammatory mediators in the arterial wall [40]. Results from our recent studies illustrate a pro-inflammatory role of apoptotic SMC in AAA. In experimental AAAs, we showed MCP-1 upregulation and apoptosis to occur primarily, though not exclusively, in the medial layer during early time points following aneurysm induction. Moreover, we showed *in vitro* that apoptotic SMCs release MCP-1 in large quantity [15], [32]. A similar link between SMC apoptosis and the production of pro-inflammatory cytokines has also been reported in atherosclerosis. Using a mouse atherosclerosis model, Clarke et al. demonstrated that SMC apoptosis induces MCP-1 expression, inflammatory infiltrate, and other features of plaque rupture [41]. By demonstrating the role of MCP-1 in regulation of FasL in aneurysmal tissue, our findings further underscore the multiple functionalities of MCP-1 and highlight its importance in regulating the interactions between residential SMCs and infiltrating macrophages in aneurysm, which ultimately amplifies signals that promote tissue destruction. Interestingly, the gene deletion of MCP-1 only provides a moderate protection of aneurysm development in mice [13], underscoring the complexity of pro-inflammatory signals in aneurysm.

Henderson EL et al. showed an elevation of FasL and Fas proteins as well as SMC apoptosis in human AAA segments [25]. Moreover, other studies showed *in vitro* that membrane bound FasL is critical for efficient activation of apoptosis while secreted soluble FasL is involved in nonapoptotic activity or led to inefficient activation of apoptotic signaling pathway [24], [26]. Our finding of MCP-1- induced membrane FasL upregulation of macrophages is likely to be an important and efficient mechanism by which macrophage-induced vascular SMC apoptosis in aneurysm development.

RIP1 has been implicated in both apoptosis and programmed necrosis termed necroptosis [42], [43]. Some reports mostly from *in vitro* studies indicate that RIP1 is recruited to a death-inducing signaling complex containing Caspase-8 and Fas-associated protein with death domain (FADD) [26], [27], [43]. Mechanistically, RIP1 is thought to be critical for efficient caspase-8 activation through participating in recruitment of caspase-8, or even efficiently and directly activating caspase-8 in the absence of FADD or TNF-R1 associated death domain (TRADD) during TNF signaling [26], [44]. Moreover, in Jurkat T lymphocytes, Morgan MJ et al. reported that RIP1 is necessary for the most efficient activation of caspase-8 when treated with membrane-bound FasL [26]. Accordingly, our results show that MCP-1 priming enhanced membrane FasL expression of macrophages, and siRNA-mediated knockdown of RIP1 in vascular SMCs rendered apoptosis resistance to SMC. Furthermore, we show *in vitro* that SMCs exposed to primed macrophages contained higher levels of RIP1/Caspase 8 containing cell death complexes, and *in vivo* evidence shows that aneurysm induction increased the level of RIP1/Caspase 8 containing complexes in medial SMCs of injured arteries. Those data herein, on the basis of known functions of membrane FasL and RIP1 in apoptosis, address the critical role of FasL/Fas-Caspase8-RIP1 mediated mechanism in macrophages-induced SMC apoptosis in aneurysm. RIP1 may be also involved in SMC apoptosis induced by other aneurysm-related extrinsic apoptotic stimuli such as TNFα, or other type of cell death such as necroptosis. Since RIP1 has not been explored in vascular SMC death and aneurysm, further investigations of this molecule for its role in the pathogenesis of aneurysm remains a highly interesting subject.

In summary, we demonstrate that the contributions of MCP-1 to aneurysm development may be also through its role in modulation of pro-apoptotic capacity of macrophages in addition to its well-conceived role as a chemokine. MCP-1 priming enhances cytotoxicity of RAW264.7 macrophages through upregulation of membrane FasL. Furthermore, our findings indicate the critical role of RIP1 in SMC apoptosis induced by macrophages. The relevance of these findings to aneurysm disease was further validated by the observation in the aneurysms that medial accumulation of macrophages coincides with SMC apoptosis, and that higher levels of RIP1/Caspase 8 containing cell death complexes are in vascular SMC-rich medial layer of injured arteries compared to that in control aortas. In the elastase-induced aneurysm, MCP-1 is necessary for macrophage infiltration as well as FasL expression of infiltrated macrophages. These findings in combination with that of previous studies together demonstrate the importance of the interaction between inflammation and apoptosis in AAA development, and indicate a role for MCP-1 in this interaction. Disruption of inflammation/apoptosis interactions in AAA may open new therapeutic avenues to develop pharmacological treatment for AAA.

## Supporting Information

Figure S1 **immunohistochemical stain of aneurysmal tissue sections confirmed elevated levels of MCP-1.** Mice were subjected to aneurysm induction by elastase. Inactive elastase-treated arteries were used as control. Cross-sections harvested 7 days after surgery were stained for MCP-1. Scale bar, 200 μm. Magnification, 10X.
(TIF)

Figure S2 **Regulation of RAW macrophage phenotypes by MCP-1.** RAW macrophages were starved for 24 hours and then treated for 24 hours with or without 100 ng/ml MCP-1. The mRNA abundance of TNFα (for pro-inflammatory M1 phenotype) and Arginase I (for anti-inflammatory M2 phenotype) were analyzed by quantitative real-time PCR. Data are mean±SEM. n = 3. *p<0.05. Two-tailed Student's *t*-test.
(TIF)

Figure S3 **FasL expression in infiltrated macrophages in MCP-1 knockout aortas following aneurysm induction.** MCP-1^−/−^ mice were subjected to aneurysm induction by elastase. Cross-sections harvested 3 days after surgery were stained for FasL (red) and macrophages (CD68, green). L indicates lumen. Scale bar, 50 μm.
(TIF)
