# Supplementary figures and images for: Monocyte Chemoattractant Protein-1 (MCP-1) Regulates Macrophage Cytotoxicity in Abdominal Aortic Aneurysm
Source: PLoS One. 2014 Mar 14;9(3):e92053. doi: 10.1371/journal.pone.0092053 (PMC3954911; doi:10.1371/journal.pone.0092053)

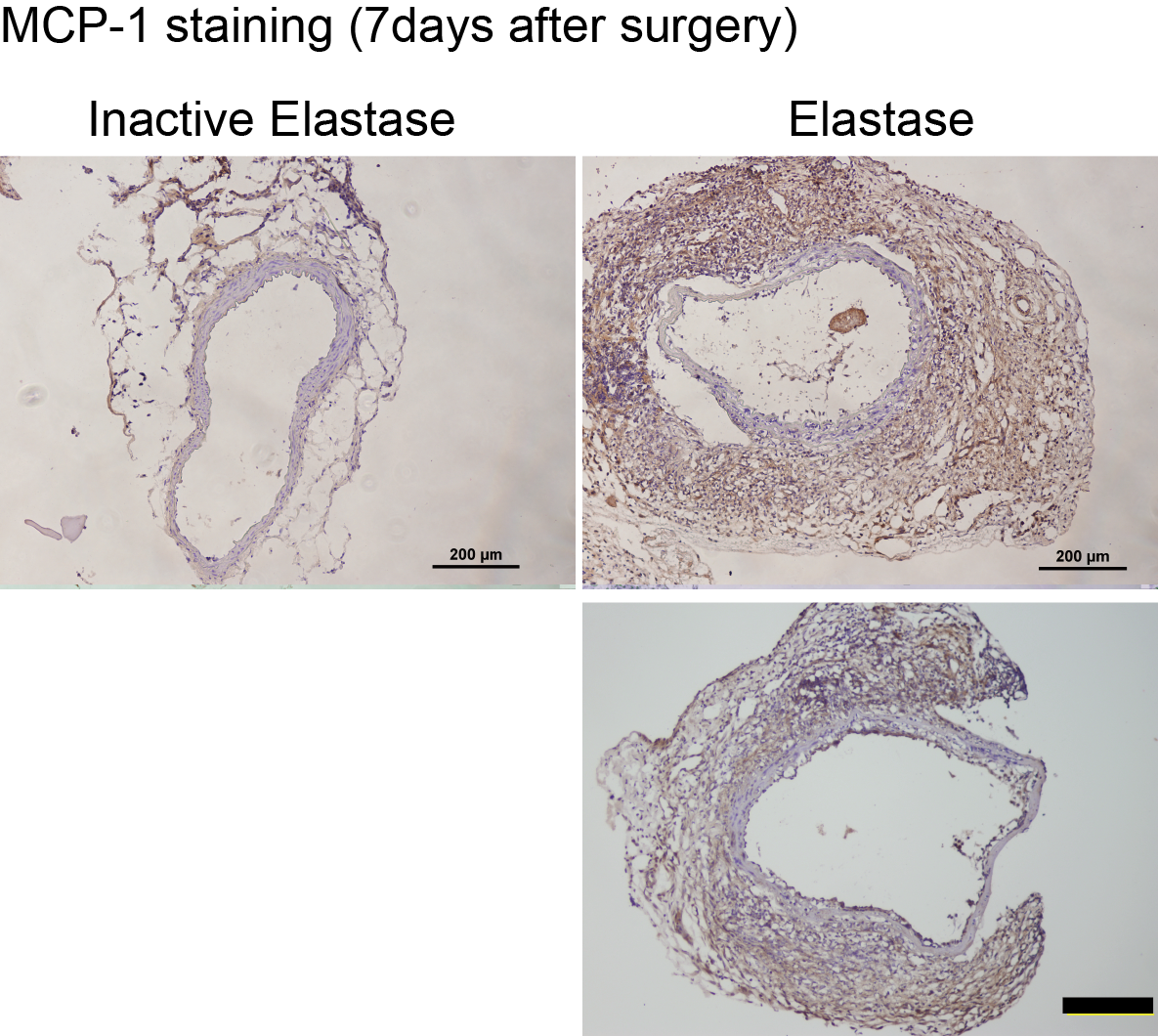

Supplement: Figure S1 — immunohistochemical stain of aneurysmal tissue sections confirmed elevated levels of MCP-1. Mice were subjected to aneurysm induction by elastase. Inactive elastase-treated arteries were used as control. Cross-sections harvested 7 days after surgery were stained for MCP-1. Scale bar, 200 μm. Magnification, 10X. (TIF) [file pone.0092053.s001.tif]

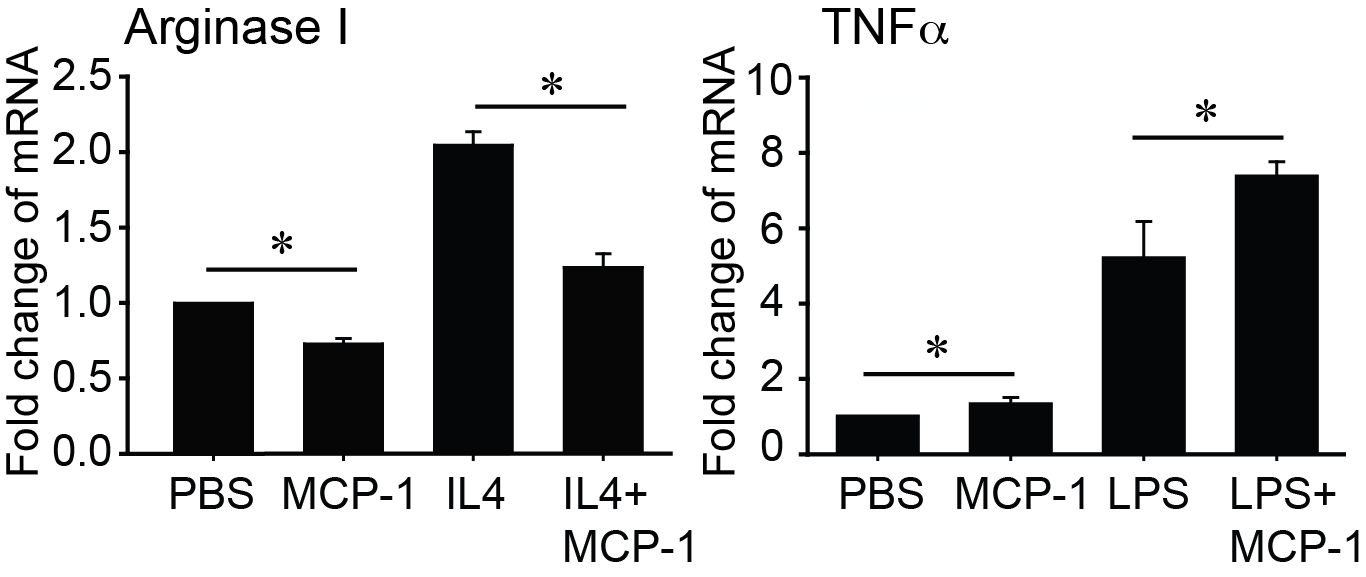

Supplement: Figure S2 — Regulation of RAW macrophage phenotypes by MCP-1. RAW macrophages were starved for 24 hours and then treated for 24 hours with or without 100 ng/ml MCP-1. The mRNA abundance of TNFα (for pro-inflammatory M1 phenotype) and Arginase I (for anti-inflammatory M2 phenotype) were analyzed by quantitative real-time PCR. Data are mean±SEM. n = 3. *p<0.05. Two-tailed Student's t-test. (TIF) [file pone.0092053.s002.tif]

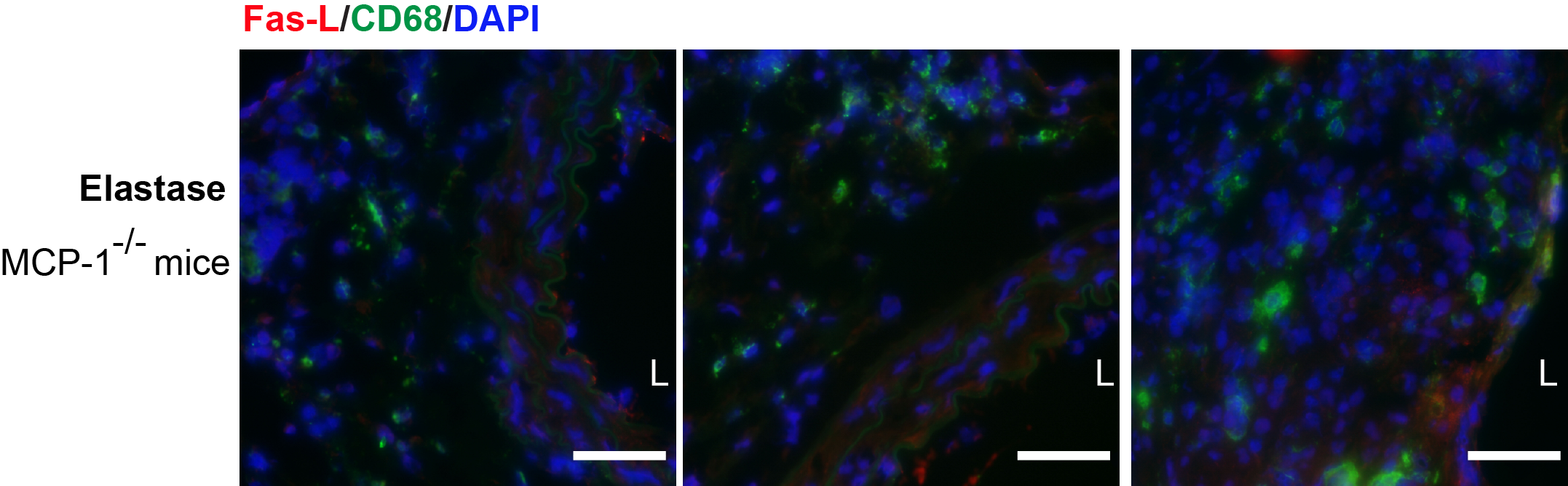

Supplement: Figure S3 — FasL expression in infiltrated macrophages in MCP-1 knockout aortas following aneurysm induction. MCP-1−/− mice were subjected to aneurysm induction by elastase. Cross-sections harvested 3 days after surgery were stained for FasL (red) and macrophages (CD68, green). L indicates lumen. Scale bar, 50 μm. (TIF) [file pone.0092053.s003.tif]
